# Microglial Activation Is Modulated by Captopril: *in Vitro* and *in Vivo* Studies

**DOI:** 10.3389/fncel.2018.00116

**Published:** 2018-05-01

**Authors:** Keren Asraf, Nofar Torika, Ron N. Apte, Sigal Fleisher-Berkovich

**Affiliations:** ^1^Department of Clinical Biochemistry and Pharmacology, Ben-Gurion University of the Negev, Beersheba, Israel; ^2^The Shraga Segal Department of Microbiology, Immunology and Genetics, Ben Gurion University of the Negev, Beersheba, Israel

**Keywords:** Alzheimer’s disease, angiotensin II, angiotensin-converting enzyme, captopril, glial inflammation

## Abstract

The renin-angiotensin system (RAS) is an important peripheral system involved in homeostasis modulation, with angiotensin II (Ang II) serving as the main effector hormone. The main enzyme involved in Ang II formation is angiotensin-converting enzyme (ACE). ACE inhibitors (ACEIs) such as captopril (Cap) are predominantly used for the management of hypertension. All of the components of the RAS have also been identified in brain. Centrally located hormones such as Ang II can induce glial inflammation. Moreover, in Alzheimer’s disease (AD) models, where glial inflammation occurs and is thought to contribute to the propagation of the disease, increased levels of Ang II and ACE have been detected. Interestingly, ACE overexpression in monocytes, migrating to the brain was shown to prevent AD cognitive decline. However, the specific effects of captopril on glial inflammation and AD remain obscure. In the present study, we investigated the effect of captopril, given at a wide concentration range, on inflammatory mediators released by lipopolysaccharide (LPS)-treated glia. In the current study, both primary glial cells and the BV2 microglial cell line were used. Captopril decreased LPS-induced nitric oxide (NO) release from primary mixed glial cells as well as regulating inducible NO synthase (iNOS) expression, NO, tumor necrosis factor-α (TNF-α) and induced interleukin-10 (IL-10) production by BV2 microglia. We further obtained data regarding intranasal effects of captopril on cortical amyloid β (Aβ) and CD11b expression in 5XFAD cortex over three different time periods. Interestingly, we noted decreases in Aβ burden in captopril-treated mice over time which was paralleled by increased microglial activation. These results thus shed light on the neuroprotective role of captopril in AD which might be related to modulation of microglial activation.

## Introduction

Alzheimer’s disease (AD) is a progressive neurodegenerative disease considered as the most common type of dementia worldwide (Stansley et al., 2012; Kettenmann et al., 2013). It is well accepted that glial-mediated inflammation contributes to the progression of the disease (Griffin, 2006; Tejera and Heneka, 2016). The AD brain is characterized by activated microglia located in close vicinity to extra-cellular cerebral depositions of amyloid β (Aβ) aggregates and intra-cellular tau-associated neurofibrillary tangles (NFTs; Heneka et al., 2014). As activated microglia are responsible for brain homeostasis, they mediate the innate immune response in the central nervous system (CNS; Tejera and Heneka, 2016). Microglia assume a variety of functions, ranging from the release of inflammatory mediators to phagocytosis (Mandrekar-Colucci and Landreth, 2010; Tejera and Heneka, 2016). Thus, microglial reactions to pathological conditions may result in a detrimental inflammatory responses leading to neurodegeneration (Griffin, 2006; Heneka et al., 2014). For example, glial cytokine production plays crucial roles in the chronic and self-sustained inflammatory cycles seen in AD, subsequently leading to neuronal dysfunction (Griffin, 2006; Glass et al., 2010). High levels of pro-inflammatory cytokines, such as interleukin-1β (IL1β) and tumor necrosis factor-α (TNF-α), were observed in the cerebrospinal fluid (CSF) and brains of AD patients (Tarkowski et al., 1999; López González et al., 2016). Reactive oxygen species (ROS), nitric oxide (NO) and elevated levels of inducible nitric oxide synthase (iNOS) enzyme, originating from resident CNS glial cells, are also observed during AD (Heneka et al., 2014). It is well established that excessive amounts of NO in the brain can shift its role from physiological neuromodulator to neurotoxic factor (Jonnala and Buccafusco, 2001). Moreover, peroxynitrite was shown to enhance Aβ peptide aggregation, leading to amyloid plaque formation via nitration of Aβ peptide residues (Kummer et al., 2011). A direct interaction between Aβ proteins and TNF-a type 1 receptor (TNFR1) was reported to stimulate inflammatory cascades leading to neuronal apoptosis (Li et al., 2004).

Over the last 25 years, anti-inflammatory agents were suggested for blocking the complement system activation in AD, induced by Aβ peptides (Breitner et al., 1995; McGeer et al., 1996). Epidemiological studies have shown various degrees of benefit from prolonged consumption of NSAIDs on the onset of AD and symptomatic severity (McGeer et al., 1996). Other prospective randomized controlled trials in adults with normal cognition or mild cognitive impairment indicated no convincing evidence for the efficacy of pharmacologic intervention with NSAIDs in reducing the risk for dementia or improving cognition (Fink et al., 2018). Interestingly, recent studies suggest a lag time of 10 and possibly 20 years as opportunity for treating AD patients with anti-inflammatory drugs prior to clinical diagnosis in order to ameliorate or prevent the disease (McGeer et al., 2016).

There is evidence showing that the brain renin angiotensin system (RAS) is associated with the development of neurodegenerative diseases via a process involving peripheral and central inflammation (Saavedra, 2012, 2016). The classical RAS can be described as a hormone system which mediates blood pressure and body fluid metabolism regulation through the main effector peptide, angiotensin II (Ang II; Skrbic and Igic, 2009). Widespread neuronal injury following glial activation by Ang II, unregulated inflammation, oxidative stress and Aβ production have been reported (Zhang et al., 2010; Zhu et al., 2011; Wang et al., 2014; Faraco et al., 2016; Torika et al., 2017).

Active Ang II is produced upon cleavage of the angiotensinogen precursor protein (Wright et al., 2008). The main enzyme involved in Ang II formation from the non-active peptide angiotensin I is angiotensin-converting enzyme (ACE). ACE was identified with other RAS components in the brain (McKinley et al., 2003; Wright et al., 2008). Moreover, elevated levels of brain ACE have been observed during AD progression (Arregui et al., 1982; Barnes et al., 1991; Savaskan et al., 2001). ACE inhibitors (ACEIs) were shown to reduce glia-induced inflammation (Hou et al., 2008; Dong et al., 2011). Although ACEIs are widely prescribed for the treatment of cardiovascular disorders, diabetes and metabolic syndrome, limited clinical studies investigated the anti-inflammatory effects of these agents in humans (Zanchetti and Elmfeldt, 2006; Savoia and Schiffrin, 2007; Kaur et al., 2015). Recent clinical studies conducted by de Oliveira et al. (2014) with perindopril and captopril found beneficial effects for such pharmacological treatment in terms of cognitive decline in late onset-AD patients. Moreover, treatment with captopril resulted in reduced amyloidogenic processing of the amyloid precursor protein (APP) and ROS levels in the hippocampus of Tg2576 AD mice (AbdAlla et al., 2013).

In the present study, we investigated the effects of captopril, a potent ACEI, administered across a wide range of concentrations, on inflammatory mediators released by lipopolysaccharide (LPS)-induced glia. Both primary glial cells and the BV2 microglial cell line were used in these studies. Targeting microglia with LPS is a well-known model for understanding the interplay between infection and neuroinflammation associated with microglial activation in brain neurodegenerative diseases (Banks and Robinson, 2010). It is well established that LPS-induced acute systemic inflammation, via stimulation of toll-like receptors 4 (TLR4) expressed on innate immune cells, can lead to lasting changes in neuroimmunomodulation and behavior (Saavedra, 2012). Actually, in the CNS, all cell types express TLRs, however, microglia express the whole repertoire and TLR4 selectively (Pardon, 2015). Interestingly, Ang II, LPS and Aβ peptides share a common mechanism for microglial activation which involves the activation of TLR (Buchanan et al., 2010; Pardon, 2015; Winklewski et al., 2016). Moreover, we considered the time-dependent effects of intranasally administrated captopril on AD-associated pathological features, gliosis and Aβ aggregation, in the brains of five familial AD mice (5XFAD). The 5XFAD mice co-express mutations in the APP and presenilin 1 (PS1) genes, which in time lead to early expression of AD-associated brain pathological features (Oakley et al., 2006). In addition to Aβ lesions and gliosis that begin to develop at 8 weeks of age in the brains of these mice, this model is one of few AD mouse models that also display cholinergic neuronal loss in different brain regions as the mice age (Yan et al., 2018).

## Materials and Methods

### Cell Culture

#### BV2 Microglial Cells

BV2 murine microglial cell line was provided by Professor Rosario Donato (Dep. of Experimental Medicine and Biochemical Sciences, University of Perugia, Italy). Cells were maintained at humified atmosphere of and 37°C and 5% CO_2_ in RPMI-1640 medium with 10% fetal calf serum (FCS), penicillin/streptomycin (100 U/ml and 100 μg/ml, respectively) and 4 Mm of L-glutamine. For experiments, cells were cultured in 24- and 6-wells plates at a density of 3 × 10^5^ and 1 × 10^6^ cells per well, respectively. Following over-night incubation, serum free medium (SFM) was added for 4 h and additional 24 h incubation with SFM containing 10 mM HEPES, 0.1% bovine serum albumin (BSA) and drug treatments was performed.

#### Primary Rat Neonatal Mixed Glial Cells Culture

Rat primary mixed glial cells cultures of astrocytes and microglia were obtained from the whole brain of neonatal (0–24 h age) Wistar rats, according to previous protocols (Brenner et al., 1992; Torika et al., 2016). Briefly, cells were harvested following meninges removal and mesh on a nylon sieves of 60 μm pore size and seeded in poly-l-lysine- coated- 24-well plates at a concentration of 1 × 10^6^ cells per well. Cells were mentioned in high glucose DMEM medium supplemented with 10% FCS, penicillin/streptomycin (100 U/ml and 100 μg/ml, respectively), 0.2 mM L-glutamine and 100 U/ml insulin. Cells were grown at humified atmosphere of and 37°C and 5% CO_2_ for 21 days, medium was replaced twice a week. For experiments, SFM was added for 4 h and replace with supplemented SFM with 10 mM HEPES, 0.1% BSA and drug treatments for 24 h.

Culture treatments included LPS from *Escherichia coli*, captopril and actinomycin D, all purchased from Sigma-Aldrich (St. Louis, MO, USA).

### Cell Viability Assay (XTT)

Cell Proliferation Kit (XTT; Biological Industries, Kibbutz Beit-Haemek, Israel) was used for determination of cells viability, according to the manufacturer’s instructions. The spectrophotometric analysis of the total formazan content was performed by using a microplate reader (model 680, Bio-Rad, Hercules, CA, USA), absorbance measured at 450 nm as previously described (Kwiecińska et al., 2012).

### Measurement of Nitrite Production (Griess Reaction)

NO production was determined by measuring the nitrite content in the supernatant of the cell culture as described previously (Zhu et al., 2010). An equal volume (100 μl) of supernatants and griess reagent (Sigma–Aldrich, St. Louis, MO, USA) were incubated for 15 min, at room temperature and light avoided atmosphere. The spectrophotometric analysis of the total nitrite content was performed by using a microplate reader (model 680, Bio-Rad, Hercules, CA, USA), absorbance measured at 540 nm. The nitrite concentration was determined using sodium nitrite as a standard (0–50 μM). Nirite levels were normalized to cell count.

### TNF-α and Interleukin 10 (IL-10) Proteins Assay by Enzyme-Linked Immunosorbent Assay (ELISA)

Supernatants TNF-α and IL-10 levels in the medium were assayed using ELISA kits (BD Biosciences, San Diego, CA, USA) according to the manufacturer’s instructions.

### Western Blot Analysis

Whole cell lysates were obtained using lysis buffer containing protease and phosphatase cocktail (Stratech Scientific LTD., UK). Samples were separated on 7.5% sodium dodecyl sulfate-polyacrylamide gel electrophoresis (SDS-PAGE), transferred to nitrocellulose membranes and blocked using 4% BSA. Overnight incubation at 4°C with specific rabbit anti-iNOS antibody (1:1000, Cayman Chemicals, Ann Arbor, MI, USA) was performed. Then, membranes were incubated with donkey anti-rabbit antibody (1:10,000, GE Healthcare, Buckinghamshire, UK) for 90 min at room temperature. The bands were visualized using enhanced chemiluminescence (ECL) solution (according to the manufacturer’s instructions) and exposure to X-ray film (Fuji medical X-ray film, FujiFilm). Computerized image analysis system (EZ Quant-Gel 2.2, EZQuant Biology Software Solutions Ltd., Israel) was used for bands analysis. Protein load was normalized by β-actin protein level measurements using membrane exposure to mouse anti-β-actin antibody (1:4000, Sigma-Aldrich) and horseradish peroxidase-conjugated goat anti-mouse antibody (1:20,000, Jackson ImmunoReaserch Inc., West Grove, PA, USA).

### Mice

The five familial AD mouse model (5XFAD) was used for animal experiments. 5XFAD mice express total of five familial AD (FAD) mutations, three mutations in the human APP695 gene (Swedish K670N, M671L; Florida I716V and London V717I) and two mutations in the human presenilin-1 gene (PSEN-1; M146L, L286V). C57BL/6 wild type (WT) mice (Harlan, Jerusalem, Israel) were reproduced with hemizygous 5XFAD mice. DNA tail polymerase chain reaction (PCR) for 3 weeks-old neonatal mice was used for detection of the human APP gene and dividing mice into WT or 5XFAD groups. Cages temperature and humidity conditions were set to 22 ± 2°C and 65 ± 5%, respectively. Mice were kept in 12 h light/dark cycle and available food/water supply conditions. For experiments, mice of both genders were randomly divided into three groups: (1) the control group included WT mice that were treated with 5 mg/kg/day of captopril (WT+Cap; *n* = 11 mice; 6 females/5 males); (2) 5XFAD mice that were treated with 5 mg/kg/day of captopril (5XFAD+Cap; *n* = 11; 5 females/6 males); and (3) 5XFAD mice that were treated with the vehicle (saline; 5XFAD+saline; *n* = 11; 6 females/5 males). Intranasal administration (2 μl drop to each nostril) of the solutions started when mice reached 8 weeks-old and lasted for 8 weeks. Surgical and experimental procedures were approved by the Institutional Animal Care and Use Committee of Ben-Gurion University of the Negev (Beer Sheeba, Israel; approval number: IL-30-08-2011-15, IL-55-09-2016).

### Immunohistochemistry

Cardiac perfusion was performed in ketamine/Xylazine Hydrochloride anesthetized mice as previously described (Torika et al., 2016; Asraf et al., 2017). Brains were then removed and the two separated hemispheres were incubated in cold 4% paraformaldehyde (PFA) solution (4°C, overnight). Hemispheres were transferred into 30% sucrose solution for 48 h followed by −80°C freezing in molds filled with tissue adhesive (O.C.T compound Tissue-Tek, Torrance, CA, USA). Brain tissues were sliced into 40 μm thick sagittal sections by cryostat (Leica, Germany) and then rinsed in 0.05% PBS/Tween 20 solution followed by another rinsing in 0.5% PBS/Triton X-100 solution. Primary antibody diluting buffer (GBI Labs, Bothell, WA, USA) was used for blockage of non-specific binding. Immunostaining for Aβ and CD11b proteins was performed using 2 h incubation with rabbit anti-human Aβ antibody (1:250, gift from Prof. Alon Monsonego, the Shraga Segal Department of Microbiology and Immunology, faculty of Health Sciences and the National institute of Biotechnology in the Negev, Ben-Gurion University of the Negev, Beer-Sheeba, Israel) and rat anti-mouse/human CD11b antibody (1:25, Biolegend) followed by incubation with the corresponding secondary antibodies, Cy3-conjugated donkey anti-rabbit IgG (1:1000, Jackson ImmunoResearch Laboratories, USA) and Alexa flour 488-conjugated goat anti-rat IgG (1:250, Jackson ImmunoResearch Laboratories, USA), respectively. Mounting medium with DAPI (Vector Labs, USA) was used for cells nuclei staining. Confocal images at a 1024 × 1024-pixel resolution with ×10 objective were obtained using the Olympus FluoView FV1000 confocal microscope (Olympus, Hamburg, Germany).

### Image Analysis

The threshold function in ImageJ software (version 1.40C, NIH, Bethesda, MD, USA) was used for quantification of the area stained for Aβ and CD11b proteins. Five cortical sections of each mouse were analyzed for the indicated proteins. The fluorescence intensity measured for the WT mice group was used as the baseline intensity.

### Statistics

Results are presented as the mean ± SEM. The statistical differences between the experimental groups were assessed by one-way analysis of variance (ANOVA) followed by *post hoc* multiple comparison test (Tukey–Kramer Multiple Comparison Test). Statistical significance was considered at *p* < 0.05.

## Results

### Captopril Treatment Does Not Show Any Cytotoxic Effect in BV2 Microglial Cells

**Figure 1:**
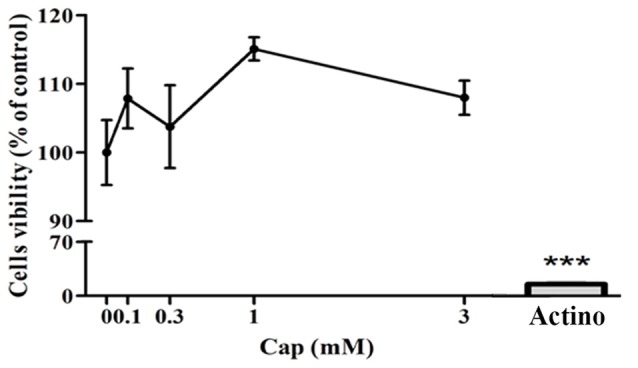
Captopril treatment does not affect BV2 microglial cell viability. BV2 cells were incubated in 96-well plates in the presence or absence of captopril (Cap; 0.1, 0.3, 1 or 3 mM) or actinomycin D (Actino; 0.25 μg/ml) for 24 h. XTT solution was added and viability was assessed using an enzyme-linked immunosorbent assay (ELISA) reader. Results are presented as means ± SEM of two independent experiments (*n* = 8). One-way ANOVA and a Tukey–Kramer multiple comparison test were used to determine statistical significance. ***p < 0.001 vs. control (non-treated cells).

We first investigated the possible cytotoxic effect of a 24 h captopril treatment on the BV2 microglial cell line using the XTT assay (Figure 1: *F*_(5,18)_ = 123, *p* < 0.0001). The effect of captopril was compared to that of the already known cytotoxic drug actinomycin D. As indicated in Figure 1, while 0.25 μg/ml actinomycin D reduced cell viability by 95%, as compared to non-treated cells (control), captopril (0.1–3 mM) did not show any cytotoxic effect.

### Captopril Dually Regulates NO Production by LPS-Treated BV2 Microglia

**Figure 2:**
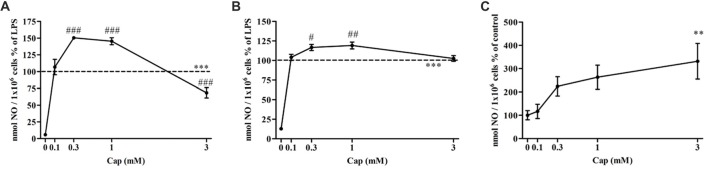
Captopril treatment differentially modulates lipopolysaccharide (LPS)-induced nitric oxide (NO) release from BV2 microglia cells. BV2 microglia were incubated in 24-well plates in the presence of 7 ng/ml **(A)** or 100 ng/ml **(B)** LPS alone or with captopril (Cap; 0.1, 0.3, 1 or 3 mM) for 24 h. 0 mM captopril represent control sample, non-treated cells. Thereafter, supernatants were analyzed for nitrite levels using the Griess reaction. Nitrite levels were normalized to cell counts. Dashed lines represent the LPS value (normalized as 100%). **(C)** NO levels measured in non-stimulated BV2 cells treated with captopril (Cap; 0.1, 0.3, 1 or 3 mM) for 24 h, presented as % of control (0 mM Cap). Results are presented as means ± SEM of three-four independent experiments (**(A)** *n* = 30, **(B,C)** *n* = 24). Statistical significance was determined using one-way ANOVA and a Tukey–Kramer multiple comparison test. **p < 0.01, ***p < 0.001 vs. control non-treated cells (0 mM Cap); ^#^*p* < 0.05 vs. LPS; ^##^*p* < 0.01 vs. LPS; ^###^*p* < 0.001 vs. LPS.

We investigated the effect of captopril on NO production levels by BV2 cells treated with two different LPS doses (7 and 100 ng/ml). As shown in Figure 2A, a significant increase in NO production was observed following 7 ng/ml LPS treatment of BV2 microglia, when compared to control non-treated cells. Treatment of BV2 cells with 7 ng/ml LPS and low doses of captopril (0.3 and 1 mM) resulted in 50% and 45% increased NO production, respectively, as compared to cells induced with LPS alone (Figure 2A: *F*_(5,96)_ = 160.8, *p* < 0.0001). Higher dose of captopril (3 mM) reduced LPS-induced NO levels in the BV2 microglial cell line by more than 40% (Figure 2A). Low captopril doses of 0.3 and 1 mM also increased NO production levels by 16% and 19%, respectively, when compared to 100 ng/ml LPS alone (Figure 2B: *F*_(5,90)_ = 107.8, *p* < 0.0001). As shown in Figure 2C, captopril increased basal NO production by 3-fold, compared with control (Figure 2C: *F*_(4,132)_ = 4.488, *p* < 0.0001).

### Captopril Dually Regulates TNF-α (Pro-inflammatory) and IL-10 (Anti- inflammatory) Production From LPS-Treated BV2 Microglial Cells

**Figure 3:**
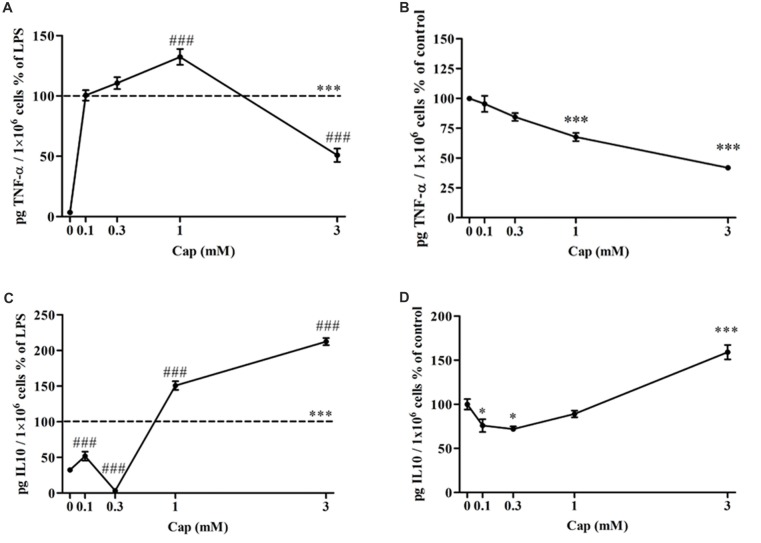
Captopril serves a dually regulates TNF-α and IL-10 production by LPS-treated BV2 microglial cells. BV2 microglial cells were incubated for 24 h with 7 ng/ml LPS alone or in the presence of captopril (Cap; 0.1, 0.3, 1 or 3 mM) **(A,C)**. Captopril (0 mM) represent control sample, non-treated cells. Supernatants were analyzed for TNF-α **(A,B)** and IL-10 **(C,D)** levels using an ELISA kits and normalized to cell counts. Dashed lines represent the LPS value (normalized as 100%). TNF-α **(B)** and IL-10 **(D)** levels measured in non-stimulated BV2 cells treated with captopril (Cap; 0.1, 0.3, 1 or 3 mM) for 24 h, presented as % of control (0 mM Captopril). Results are presented as means ± SEM of two-four independent experiments (**(A)** *n* = 30, **(B)** *n* = 24, **(C)** *n* = 32, **(D)** *n* = 30). Statistical significance was determined using one-way ANOVA and a Tukey–Kramer multiple comparison test. *p < 0.05 vs. control non-treated cells; ***p < 0.001 vs. control non-treated cells; ^###^*p* < 0.001 vs. LPS.

The effect of captopril on the secretion of pro-inflammatory TNF-α, and anti-inflammatory IL-10 from BV2 microglial cells was assessed (Figure 3). TNF-α levels were significantly increased by more than 97% in 7 ng/ml LPS-treated BV2 microglial cells, as compared to controls (Figure 3A: *F*_(5,283)_ = 202.4, *p* < 0.0001). Incubation with low doses of captopril (0.1 and 0.3 mM) did not affect LPS-induced TNF-α production levels. When administered at a 1 mM concentration, captopril elicited a 32% increase over that observed following treatment with LPS alone (Figure 3A). By contrast, captopril provided at 3 mM abrogated the effect of LPS on TNF-α production by about 50% (Figure 3A). Basal TNF-α levels were significantly reduced upon captopril treatment (Figure 3B: *F*_(4,100)_ = 38.78, *p* < 0.0001). 7 ng/ml LPS treatment significantly increased the IL-10 production levels when compared to controls (Figure 3C: *F*_(5,63)_ = 236.7, *p* < 0.0001). Treatment with lower doses of captopril (0.1 and 0.3 mM) reduced IL-10 release by LPS-treated cells (Figure 3B). However, a robust increase in IL-10 production was observed following treatment with higher doses of captopril (1 and 3 mM). Captopril alone significantly affected IL-10 production levels in non-stimulated BV2 cells (Figure 3D: *F*_(4,57)_ = 35.9, *p* < 0.0001). While low doses of 0.1 and 0.3 mM captopril reduced IL-10 production by approximately 23%, compared to control cells, 3 mM captopril increased its production by 59% (Figure 3D).

### Captopril Decreases NO and TNF-α Production Levels by LPS-Treated Neonatal Mixed Glial Cells

**Figure 4:**
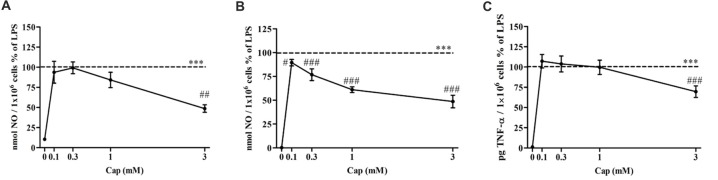
Captopril attenuates NO and TNF-α production levels in LPS-treated primary mixed glial cells. Primary neonatal mixed glial cells were incubated in 24-well plates in the presence of 100 ng/ml **(A)** or 0.5 μg/ml **(B,C)** LPS alone or with captopril (Cap; 0.1, 0.3, 1 or 3 mM) for 24 h. Captopril (0 mM) represent control sample, non-treated cells. Supernatants were analyzed for nitrite and TNF-α levels and normalized to cell counts. Dashed lines represent the LPS value (normalized as 100%). Results are presented as means ± SEM of two-three independent experiments (**(A)** *n* = 24, **(B)** *n* = 36, **(C)** *n* = 24). Statistical significance was determined using one-way ANOVA and a Tukey–Kramer multiple comparison test. ***p < 0.001 vs. control non-treated cells (0 mM Cap); ^#^*p* < 0.05 vs. LPS; ^##^*p* < 0.01 vs. LPS; ^###^*p* < 0.001 vs. LPS.

The effect of captopril on NO production levels by primary mixed glial cells was investigated in 100 ng/ml and 0.5 μg/ml LPS-treated cells. Treatment with 100 ng/ml (Figure 4A) and 0.5 μg/ml (Figure 4B) LPS resulted in robust induction of NO production levels. 3 mM captopril treatment significantly reduced the NO production levels by approximately 50% compared to 100 ng/ml LPS-treated cells (Figure 4A: *F*_(5, 51)_ = 14.55, *p* < 0.0001). While lower captopril doses (0.1 and 0.3 mM) reduced the LPS (0.5 μg/ml)-induced NO production by 11% and 24%, respectively, higher doses of the inhibitor (1 mM and 3 mM) resulted in 40% and 50% reduction of NO production by LPS-treated primary mixed glial cells, respectively (Figure 4B: *F*_(5,205)_ = 293.7, *p* < 0.0001). Moreover, 30% reduction in TNF-α production levels were observed following 3 mM captopril treatment of 0.5 μg/ml LPS-treated primary mixed glial cells (Figure 4C: *F*_(5,92)_ = 101.6, *p* < 0.0001).

### Dual Effect of Captopril on LPS-Induced iNOS Protein Expression Levels in BV2 Microglial Cells

**Figure 5:**
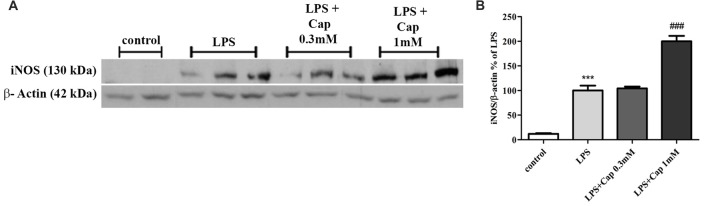
Captopril (at low concentrations) increases LPS-induced inducible NO synthase (iNOS) expression in BV2 microglial cell line. Cells were incubated for 24 h in the presence or absence of LPS (7 ng/ml) alone or with captopril (Cap; 0.1, 0.3, 1 or 3 mM). Thereafter, whole cell lysates were obtained and proteins were separated by SDS-PAGE. Levels of iNOS protein (130 kDa) were determined relative to β-actin (42 kDa) levels by Western analysis using target-specific primary antibodies. Representative blots **(A)** are shown. Results are presented in graph **(B)** as means ± SEM of two independent experiments (*n* = 18). One-way ANOVA and a Tukey–Kramer multiple comparison test were used to determine statistical significance. ***p < 0.001 vs. control non-treated cells; ^###^*p* < 0.001 vs. LPS.

We previously showed a 50% reduction in iNOS expression upon treatment with higher concentrations of captopril in LPS-treated BV2 cells, as compared to cells treated with LPS (7 ng/ml) alone for 24 h (Torika et al., 2016). In contrast, iNOS expression was amplified 2-fold by adding 1 mM captopril over the level of enzyme expression in cells treated solely with LPS (Figure 5: *F*_(3,15)_ = 96.22, *p* < 0.0001).

### A 2-Month Intranasal Captopril Treatment Ameliorates Gliosis and Aβ-Pathology in Cortical Layers of 5XFAD Mice

**Figure 6:**
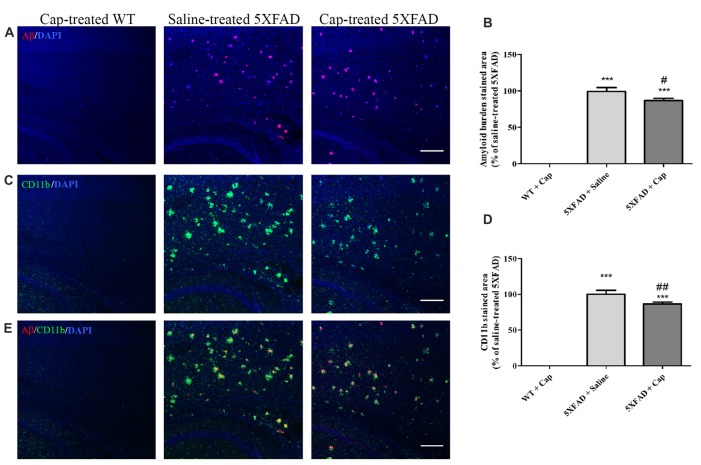
Intranasal administration of captopril reduces amyloid burden and CD11b expression in the cortex of 5XFAD mice. Eight week-old wild type (WT) or 5XFAD mice were treated intranasally with either saline or captopril (cap; 5 mg/kg/day) for 2 months. At the end of treatment, the mice were anesthetized and cardiac perfusion with cold PBS was performed. Brains were fixed in 4% paraformaldehyde (PFA) and 30% sucrose solutions. Then, 40 μm-thick brain sagittal sections were stained for Aβ (red) and CD11b (green) proteins using target-specific antibodies and counterstained with mounting solution containing DAPI (blue). Representative cortical layers from the three mice groups are presented. The experiment included 11 mice per group (*n* = 33). The calculated average sums of Aβ- **(A,B)** and CD11b-stained **(C,D)** cortical areas are represented as mean percentage ± SEM of the stained area in the saline-treated group in five repeats. Merged images of anti-Aβ and anti-CD11b staining are presented **(E)**. One-way ANOVA and a Tukey–Kramer multiple comparison test were used to determine statistical significance. The scale bar is 200 μm. ***p < 0.001 vs. WT+cap; ^#^*p* < 0.05 vs. 5XFAD+saline; ^##^*p* < 0.01 vs. 5XFAD+saline.

The effects of captopril, given at a clinically relevant dose via intranasal administration procedure, on CD11b expression and amyloid burden in 5XFAD mice cortex were studied, as was brain immunohistochemistry (Figure 6). Cortical sections of 4 month-old WT mice showed low CD11b expression (Figures 6C,D), with no Aβ formation (Figures 6A,B). By contrast, cortical section of age-matched 5XFAD mice exhibited increased levels of Aβ plaques (Figures 6A,B) and the CD11b marker (Figures 6C,D), when compared to WT-treated mice. Intranasal administration of 5 mg/kg/day captopril significantly reduced the areas stained for CD11b (Figures 6C,D: *F*_(2,43)_ = 515.8, *p* < 0.0001) and Aβ (Figures 6A,B: *F*_(2,43)_ = 272.3, *p* < 0.0001) proteins in the cortex of 4 month-old 5XFAD mice, when compared to saline-treated 5XFAD mice brain sections.

### Different Time-Dependent Effects of Intranasally Administered Captopril on Gliosis and Aβ Pathology in the Cortical Layers of 5XFAD Mice

We compared the effects of intranasal exposure of mice to captopril (5 mg/kg/day) for different periods of time on gliosis and amyloid burden expression in the cortical areas of 5XFAD mice (Table 1). As indicated in Table 1, the decrease of Aβ burden in captopril-treated mice over time (125.5%–75.1%) was paralleled by increased microglial CD11b expression (67.8%–101%).

**Table 1:** Overview of the time-dependent effects of intranasal captopril on gliosis and Aβ expression in 5XFAD mice cortical areas.

Captopril dosage	Treatment period	Amyloid burden stained area (% of Saline-treated 5XFAD)	CD11b stained area (% of Saline-treated 5XFAD)
5 mg/kg/day	3.5 weeks	125.48 ± 14.66	67.82 ± 3.61***
5 mg/kg/day	2 month	87.49 ± 3.15*	86.52 ± 2.68**
5 mg/kg/day	7 month	75.08 ± 4.81***	101.02 ± 5.34

5XFAD mice were treated intranasally with either captopril or saline for different times (3.5 weeks, 2 months or 7 months). At the end of the experiment, the mice were anesthetized and cardiac perfusion using cold PBS was performed. Brains were removed and 40 μm-thick sagittal sections were stained for CD11b and Aβ proteins using target-specific antibodies. The averaged sum of the areas stained for CD11b and amyloid burden in the cortex of 5XFAD mice treated with captopril was compared to that one measured in age matched 5XFAD mice treated with saline. The calculated average sums of Aβ- and CD11b-stained cortical areas are represented in the table as mean percentage ± SEM of the stained area in the saline-treated group. One-way ANOVA and a Tukey–Kramer multiple comparison test were used to determine statistical significance. *p < 0.05 vs. saline-treated 5XFAD mice, **p < 0.01 vs. saline-treated 5XFAD mice, ***p < 0.001 vs. saline-treated 5XFAD mice.

## Discussion

Although microglia comprise only 10% of the CNS cell population, much of the innate immune response in the CNS is mediated by these cells (DiSabato et al., 2016). The microglial inflammatory response can be mimicked by the use of LPS endotoxin, which triggers microglia to secrete a wide variety of inflammatory cytokines (Pardon, 2015).

In this study, a robust inflammatory response by BV2 microglial cells was observed following LPS treatment, and resulted in the enhanced release of TNF-α, and NO, as well as elevated levels of iNOS expression (Figures 2–5). The present study also provides evidence for the first time that ACE inhibition by captopril serves a dual role in microglia-mediated neuroinflammation.

Dual regulation of neuroinflammation was also observed by us with kinins. Stimulation of the bradykinin 2 receptor (BK2R) enhanced glial inflammation in a manner that was blocked by BK2R antagonist. By contrast, a BK 1 receptor (BK1R) agonist attenuated the glial inflammatory response (Levant et al., 2006). This may partially explain the dual effects of captopril shown in the present study. In addition to intervening in Ang II metabolism, ACE can also metabolize bradykinin (BK) to form a non-active peptide (Camargo et al., 2012; Igic and Skrbic, 2014). It is well established that BK has high affinity to BK2Rs, while B1Rs are specialized for responding to BK metabolites (Moreau et al., 2005). ACE inhibition interferes with BK breakdown and prolongs its half-life (Igic and Skrbic, 2014). Based on the above, it is assumed that different captopril doses lead to differential BK1R/BK2R activation balance.

Previously, Bhat et al. (2016) showed the anti-inflammatory effects of perindopril (1 nM–1 μM), a centrally active ACEI, in LPS-treated glial cell culture. RAS intervention by perindopril ameliorated astrocytic and glial activation and reduced the production of TNF-α and oxidative stress markers and in parallel, elevated IL-10 levels (Bhat et al., 2016). Furthermore, 1 mM captopril was reported to suppress the production of the pro-inflammatory cytokine interleukin 12 (IL-12) by human peripheral blood mononuclear cells (Constantinescu et al., 1998).

In contrast to the proposal that captopril acts as an anti-inflammatory, some data argues that this agent mediates the opposing effect at certain concentrations. For instance, Coelho dos Santos et al. (2010) showed that captopril induced inflammation in human monocytes and peripheral mononuclear cells. The authors also suggested that captopril increased the monocyte infection involved in Chagas disease by induction of interleukin 17 (IL-17) and inhibition of IL-10 production (Coelho dos Santos et al., 2010). Goel et al. (2015) studied the effects of orally administered perindopril (0.1 mg/kg) on inflammatory and oxidative stress features in spontaneous hypertensive rats (SHRs) brain. In their study, the already high levels of TNF-α, iNOS, nitrite and ROS observed in the brains of SHRs were further exaggerated following intracervical LPS administration but decreased in response to perindopril treatment (Goel et al., 2015). Similar anti- inflammatory effects and suppression of pro-inflammatory mediators were also observed in LPS-injected rats treated with captopril (1–100 mg/kg) via inhibition of NF-κB pathways (Ilieva et al., 2006; Muñoz et al., 2006).

Captopril dually regulated both NO and TNF-α secretion from BV2 cells (Figures 2, 3). An inhibitory effect of captopril on both NO and TNF-α was observed in primary mixed glial cultures comprising both microglia and astrocytes (Figure 4). The reciprocal interactions between microglia and astrocytes may be particularly important for the distinct effects observed with captopril in mixed glial cultures, as compared with microglial cell lines. Differential sensitivity of cells to captopril may be due to the presence of different types or quantities of endopeptidases or to different densities of BK and/or Ang II receptors in these cells. Different LPS concentrations were used to induce inflammation in both cell types. The dual effects of captopril, on “inflammatory molecules” release, probably do not depend on inflammation grade (Figures 2, 4).

In addition to *in vitro* anti-inflammatory properties of captopril, we demonstrated reduced amyloid burden and macrophage/microglia accumulation in the cortex of 5XFAD mice following a 2-month-long intranasal captopril treatment, when compared to age-matched saline-treated 5XFAD mice (Figure 6). Although ACE is believed to convert neurotoxic Aβ42 peptides into a shorter form of Aβ40, thought to be less toxic in AD, the manner in which ACE inhibition affects amyloid peptide forms in AD is not yet well understood (Eckman et al., 2006; Zou et al., 2007, 2013; Regenold et al., 2017). It was previously reported that a 6-month-long captopril treatment of AD mice reduced markers of amyloidogenic processing of full-length APP and resulted in slower hippocampal Aβ accumulation (AbdAlla et al., 2013). Up-regulation of APP and tau hyper-phosphorylation mediated by captopril were also reported in a recent study by the same research group (AbdAlla et al., 2015). By contrast, captopril was shown to promote Aβ42 deposits in an AD mouse model and in cell culture studies (Hemming and Selkoe, 2005; Zou et al., 2007). Other AD studies suggested that ACEIs do not alter brain Aβ levels (Hemming et al., 2007; Dong et al., 2011; Wharton et al., 2012). As the present study showed that a 2-month-long intranasal captopril treatment reduced the Aβ burden and gliosis in the 5XFAD mouse cortex, we assume that the intranasal delivery procedure employed promotes an additive beneficial effect over systemic ACEI administration. Intranasal delivery has been suggested to enhance therapeutic delivery to the brain and allow direct entry to the CNS with minimal systemic exposure (Dhuria et al., 2010). However, the mechanism by which intranasal captopril administration ameliorates the amyloid burden in the cortex of 5XFAD mice should be further examined. This effect could be mediated by mechanisms which involve elevated clearance of Aβ via phagocytic microglial cells (Doens and Fernández, 2014), variations in Aβ-degrading enzyme expression (Nalivaeva et al., 2012) or changes in the generation of Aβ peptides followed by lowered brain inflammation (Griffin, 2006).

Table 1 summarizes what we have observed (Torika et al., 2016) with respect to the effects of intranasal captopril treatment on cortical Aβ and CD11b expression in the brain of 5XFAD mice over three different time periods. Interestingly, our findings show decreased burden in captopril-treated mice over time which was paralleled by increased microglial activation. In AD patient brain, the amyloid burden is accompanied by a clustering of activated microglia around the amyloid plaques. Reduced Aβ depositions, alongside microglial activation and enhanced phagocytic ability by angiotensin-related drugs, was shown to potentially improve cognitive performance in AD mice (Tsukuda et al., 2009; Shindo et al., 2012; Torika et al., 2017). It is worth noting that reduced amyloid burden can also involve other mechanisms which are not necessarily related to changes in microglial activity. Further studies are required to conclude whether intranasally administered captopril alters Aβ-degrading enzyme expression or influences other mechanisms involved in Aβ production.

## Author Contributions

KA, NT and SF-B designed the experiments. KA, RNA and NT performed cell culture experiments. KA performed *in vivo* experiments and analyzed the data. SF-B secured funds for this work. NT and SF-B wrote the manuscript. All authors read the manuscript and approved its final content.

## Conflict of Interest Statement

The authors declare that the research was conducted in the absence of any commercial or financial relationships that could be construed as a potential conflict of interest.
